# Consequences of early postnatal benzodiazepines exposure in rats. II. Social behavior

**DOI:** 10.3389/fnbeh.2014.00169

**Published:** 2014-05-08

**Authors:** Anna Mikulecká, Martin Šubrt, Martina Pařízková, Pavel Mareš, Hana Kubová

**Affiliations:** Institute of Physiology, Academy of Sciences of the Czech RepublicPrague, Czech Republic

**Keywords:** benzodiazepines, clonazepam, social behavior, development, rats

## Abstract

Social behavior represents an integral part of behavioral repertoire of rats particularly sensitive to pharmacological and environmental influences. The aim of the present study was to investigate whether early postnatal clonazepam (CZP) exposure can induce age-dependent changes related to expression of social behavior. The drug was administered from postnatal day (P) 7 until P11 at daily doses of 0.1, 0.5 and 1.0 mg/kg i.p. We designed three experiments to assess whether exposure to CZP affects social behavior in respect to the age of rats and the test circumstances, specifically their familiarity with test conditions during adolescence (P32), social behavior in juveniles and adolescents (P18–P42) and social behavior in a resident-intruder paradigm. The frequency and duration of a various patterns of social behavior related to play and social investigation not related to play were evaluated. The results showed that CZP postnatal exposure decreased social play behavior regardless of age and familiarity or unfamiliarity of experimental environment but did not affect the social investigation *per se*. When rats were confronted with an intruder in their home cages intense wrestling and inhibition of genital investigation were found. In conclusion, these findings show that short-term CZP postnatal exposure inhibits social play behavior and alters specific patterns of social behavior in an age and environment related manner.

## Introduction

Benzodiazepine (BZD) exposure during brain development can result in persistent modification of brain functions, behavioral alterations and cognitive deficits (for rev. Gai and Grimm, 1982; Tucker, 1985; Kellogg, 1988). Enduring behavioral, biochemical and molecular effects can also occur when drugs are administered after neuronal differentiation, but before complete maturation of the central system, i.e., during the first three weeks of life in rats (Avishai-Eliner et al., 2002) or later during adolescence (Hulin et al., 2012). In spite of possible risk of adverse effects, BZDs are widely prescribed to treat anxiety, depression and insomnia, to control epileptic seizures, promote anesthesia or to induce muscle relaxation in all age groups of patients including neonate and children (for rev. Lader, 1994; Lalive et al., 2011).

Effects of BZDs are specifically mediated by their interaction with BZD receptor binding site, which modulates the efficacy of the major inhibitory neurotransmitter, γ-aminobutyric acid (GABA), at the BZD-sensitive GABA_A_ (BZD/GABA_A_) receptors. In rats, BZD/GABA_A_ receptors can be detected during the last week of gestation and they reach the adult level by the third-fourth week of life (Lippa et al., 1981). Changes of BZD/GABA_A_ receptor features were detected in animals exposed to BZDs prenatally as well as postnatally (for rev. Tucker, 1985). A recent study documented that early treatment with BZDs induces selective alteration of subunit expression. Changes at receptor level were observed also after administration of other GABA_A_ receptor ligands as barbiturates (Raol et al., 2005). In addition, administration of GABAergic drugs including BZDs during the 1st week of life increases apoptosis (Bittigau et al., 2002; Forcelli et al., 2012). Therefore changes at both molecular and cellular level can result in alteration of neuronal circuitry in the immature brain and induce functional impairment.

Despite high clinical importance, possible long-term risks of early postnatal exposure to BZDs are only rarely investigated. Some changes of behavior related to anxiety, aggression, emotional state and cognitive abilities were reported in animals exposed to BZDs from birth until weaning (P1–P21) and tested during adolescence or adulthood (File, 1986a,b, c; Schroeder et al., 1997). In these studies exposure to BZDs lasted for long time and affected several critical developmental periods including switch in BZDs receptor structure and maturation of the hippocampal formation (Garrett et al., 1990; Avishai-Eliner et al., 2002). Due to sedation and myorelexation (for rev. Tucker, 1985), long lasting administration of BZDs was found to cause undernutrition and significant growth retardation, which can negatively impact future functional abilities of exposed individuals (Smart et al., 1973). Even though adverse effects of long lasting BZD exposure can substantially affect results of developmental studies, chronic consequences of short lasting BZD exposure are sparse. File (1987) demonstrated changes of exploratory activity and emotional state (e.g., increased aggression) in adolescent animals exposed to diazepam at P1–P7 (File, 1987). Recently, studies with another modulator of GABA_A_ receptors, allopregnanolone, showed long-lasting emotional and cognitive impairment in adult animals exposed to this neurosteroid between P5 and P9 (Darbra and Pallarès, 2009; Mòdol et al., 2013). Thus available data clearly document that early modulation of GABA_A_ receptors can modify behavior at later stages of development and relate to a susceptibility to psychopathology in adulthood.

Studies on functional cosequences of early BZD exposure have focused mostly on cognitive abilities and emotional state. Alterations of social behavior are examined only sporadically despite an importance of normal social interactions for further development and life in highly organized groups. Only File’s studies have documented impairment of social interactions in animals exposed to BZDs early in the life and suggested that alteration depends on the time-course of drug exposure, dose and experimental approach (File, 1986b,c, 1987). Data are however still fragmentary.

Present study was designed to determine whether exposure to BZDs at early postnatal stage (P7–P11) which corresponds with perinatal period in humans (Dobbing and Sands, 1979; Avishai-Eliner et al., 2002; Clancy et al., 2007), affects: (1) social behavior during adolescence (P32); (2) social behavior in juveniles and adolescents (P18–P42); and (3) social behavior of rats in their homing environment at three developmental stages: middle adolescence (P32), sexual maturity (P60), and adulthood (P80). The general design of used behavioral tests was adapted from the literature (Meaney and Stewart, 1981; Fraňková and Mikulecká, 1990; Vanderschuren et al., 1995b; Mitchell and Redfern, 2005).

As a model BZD, we chose clonazepam (CZP), a partial BZD agonist, first used for treatment of certain types of seizures (Morishita, 2009). Safety and negligible adverse effects of CZP in immature rats were demonstrated before (Mikulecká et al., 2011). Data on pharmacokinetics of CZP in rats are sparse, but Hoogerkamp et al. (1996) showed that terminal half-life of CZP in adult rats is approximately 1 h. Immature organisms eliminate CZP more slowly than adults (André et al., 1986). Our previous studies demonstrated duration of both anticonvulsive and anxiolytic effects in rat pups. After single administration of CZP in a dose of 1.0 mg/kg both P7 and P12, animals were partially protected against pentylenetetrazole (PTZ)-induced seizures for 24 h (Kubová and Mareš, 1989). CZP in the same dose exhibited anxiolytic effects in P12 rats for 48 h whereas motor impairment was observed for only 3 h (Mikulecká et al., 2011). Also repeated CZP administration only minimally affected body growth and did not affect maternal attention and care (Mikulecká et al., 2014).

## Material and methods

### Animals

Male Wistar albino rats (Institute of Physiology, Academy of Sciences, Prague, *n* = 190) were used and maintained under controlled temperature (22 ± 1°C) and humidity (50 to 60%) with a 12/12 h light/dark cycle (lights on at 6:00 AM). Food and water were provided ad libitum (with the exception of the testing period). On day 5, (birth counted as day 0) the pups were randomly fostered and each litter was adjusted to ten males. At P7 the animals were marked for identification and mixed by treatment. To exclude the participation of a litter effect, only a limited number of animals from the same litter were used. They were weaned at postnatal day (P) 28. After weaning, the animals were housed in groups of 3–4 per cage. Experiments were approved by the animal Care and Use Committee of the Institute of Physiology, Academy of Sciences of the Czech Republic (v.v.i), and determined to be in agreement with the Animal Protection Law of the Czech Republic, which is fully compatible with the guidelines of the European Community Council directives 86/609/EEC.

### Drug exposure

CZP (Hoffmann–Switzerland; obtained as a gift) was suspended in one drop of Tween 80 and 1 ml of saline. Subsequently, this solution was diluted in such a way that all used doses of CZP were administered in the same volume of 5 µl/g of body weight. The doses were selected according to our previous studies on anticonvulsant effects of CZP in developing animals (Kubová and Mareš, 1989; Mikulecká et al., 2011). The doses were optimized according to results obtained in the first experiment, *social behavior in adolescence* (Experiment 1). In this experiment CZP at the doses of 0.1, 0.5 and/or 1.0 mg/kg were administered intraperitoneally for 5 consecutive days, starting from P7 to P11. In other experiments only doses of 0.5 or 1.0 mg/kg were used. Control animals received the corresponding volume of vehicle. Animals were weighted daily during the CZP exposure, on P12 and P15, and at the beginning of each experiment. Overall health condition was regularly examined.

### Experimental design

**Figure 1:**
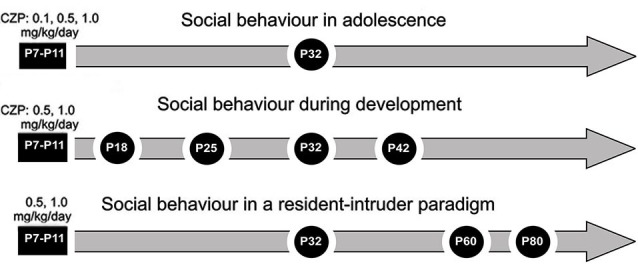
**Design/time diagram of experimental procedure: *social behavior in adolescence, social behavior during development and social behavior in a resident-intruder paradigm***. The upper part shows a timeline for the Experiment 1: *social behavior in adolescence* after 0.1, 0.5 and 1.0 mg/kg/day of CZP exposure between P7 and P11. The middle part shows a timeline for the Experiment 2: *social behavior during development* after 0.5 and 1.0 mg/kg/day of CZP exposure. The lower part shows a timeline for the Experiment 3: *social behavior in a resident-intruder paradigm*.

The experimental procedures are depicted in Figure 1. All experiments were performed in a room with constant temperature (22 ± 1°C), under low-light conditions (35–45 lx), between 9:00 AM and 3:00 PM. Animals behavior was video recorded and then analyzed using Observer, and locomotion was assessed using EthoVision (both software Noldus Information Technology). Two experimenters analyzed the video recordings simultaneously and repeatedly until both of them were able to recognize the same patterns. The patterns with duration of one second or more were counted. Then, both observers performed evaluation of recordings separately, and between-reliability was calculated (between-reliability >0.9).

#### Experiment 1: Social behavior in adolescence

Animals were tested at the age of 32 days (P32). The control group consisted of nine pairs of rats. The group exposed to CZP at a dose of 0.1 mg/kg consisted of eight pairs and those exposed to CZP at a dose of 0.5 mg/kg and 1.0 mg/kg both consisted of nine pairs. Two consecutive days before social interaction, the animals were familiarized to the experimental condition by placing them individually into the open field arena (OF) (45 × 45 × 30 cm) for 5 min. On the day of social interaction test, the rats were weighted, marked and individually isolated into small cages (21 × 27 cm) for three hours. Two rats from different litters with identical treatment (C vs. C, CZP vs. CZP) were placed into the OF arena at opposite corners and their behavior was recorded for 10 min. To reduce any lingering olfactory cues, OF was thoroughly wiped with water-moistened tissue paper after each pair tested. The frequency and the duration of the following patterns per pair of rats were observed: following/chasing (moving in the direction of or pursuing the partner that is moving away); climbing over/under; mutual sniffing; boxing (the animals stand upright facing one another and pawing toward each other); wrestling (the animals rolled over each other in rough-and-tumble play) and pinning (one of the animals is lying in supine position and the other is laying over it). These behavioral patterns were divided into *Behavior related to play* (pinning, boxing/wrestling, following/chasing) and *Behavior unrelated to play* (climbing over/under and mutual sniffing) and then analyzed separately.

#### Experiment 2: social behavior during development

Social behavior during development was studied in 10 pairs of controls and two groups of 10 pairs of animals exposed to CZP. Animals were tested for the first time at P18 and then repeatedly at P25, P32 and P42. At the beginning of each test, animals were weighted, marked and individually isolated into small cages (21 × 27 cm) for 30 min. Similar to Experiment 1, two rats from different litters with identical treatment (C vs. C, CZP vs. CZP) were placed into the OF arena at opposite corners and their behavior was recorded for 10 min. The duration of following behavioral patterns per pair was evaluated: passive contact (pups in close contact, resting, with no movement), active contacts (crawling over/under, mutual sniffing), following (one pup followed the other pup rapidly so that it almost touched it) and play fighting (the animals rolled over each other in rough-and-tumble play). Locomotion expressed as the distance travelled was assessed for both animals in the pair.

#### Experiment 3: social behavior in a resident-intruder paradigm

**Figure 2:**
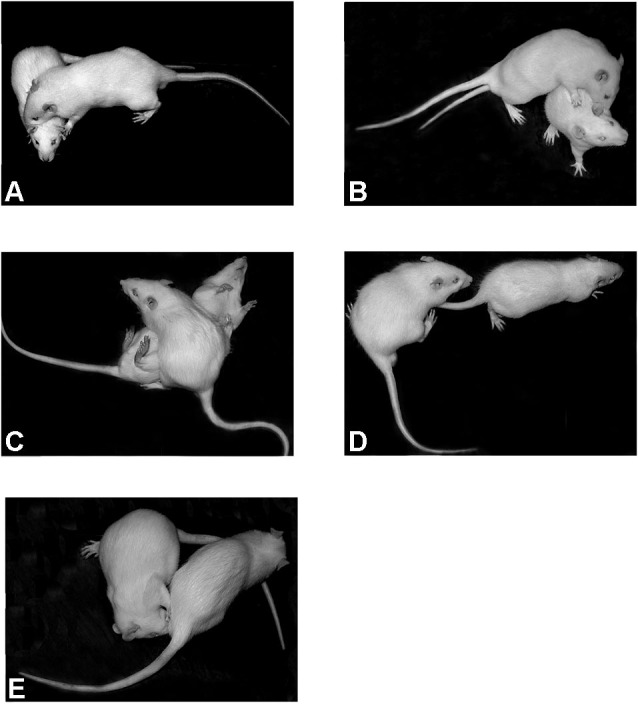
**Social behavioral patterns displayed by rats in a resident-intruder paradigm**. **(A)** pouncing, **(B)** wrestling, **(C)** pinning, **(D)** following, and **(E)** genital investigation.

Three groups of rats each consisting of 10 animals were used. Two groups were exposed to CZP at doses of 0.5 or 1.0 mg/kg and one control group was injected with saline. The animals were housed in cages containing three to four rats. Twenty four hours before testing, the animals designated as resident rats were housed individually in standard cages in order to provide territorial advantage. The rats serving as social stimuli (intruder rats), were not isolated. On the experimental day (P32), the animals in their home cages were transferred to the experimental room for an acclimatization period of 30 min before testing. Each resident was paired with an unfamiliar untreated intruder. The test started by placing an intruder in the resident home cage and their social interaction was video recorded for 10 min. After the test was completed, both resident and intruder rats were returned to their respective group cages (to re-establish each group member’s rank position within the social hierarchy). The same pairs of animals were tested at P60 and P80 following the same procedure as at P32. The following behavioral patterns were observed: pouncing (one rat soliciting the other, by attempting to nose or rub the nape of its neck); following (the pursuit of one animal by another); wrestling (rough and tumble play); pinning (the one animal stands over the exposed ventral area of the other pressing it down); genital investigation (sniffing anogenital area). Behavioral patterns were observed separately for each member of a pair (resident and intruder) except for wrestling because this pattern involved two animals concurrently performing the same behavior (Figure 2). The day following the social interaction test, locomotion of residents was assessed in the OF (see below).

### Locomotion

After 30 min of adaptation to the experimental room, a rat was placed into the left corner of the arena to explore the new environment for 5 min. Locomotion was evaluated by analyzing the track record for the distance travelled. In both Experiment 1 (*social behavior in adolescence*) and Experiment 2 (*social behavior during development*) locomotion for each rat in the pair was calculated. In Experiment 3 (*social behavior in a resident-intruder paradigm*) locomotion was assessed only for resident rats one day after social behavior test.

### Statistical analysis

The frequency and duration of social interaction patterns were evaluated. All data were presented as mean ± standard error of mean. One-way ANOVA was used to analyze data from Experiment 1 (*social behavior in adolescence*). Data from Experiment 2 (*social behavior during development*) and from Experiment 3 (*social behavior in a resident-intruder paradigm*) were analyzed with two-way ANOVA (one treatment factor and age as a repeated-measures factor). Further, *post hoc* Holm-Sidak method was used to explore significant main effects or interactions resulting from the ANOVA (SigmaStat® SPSS Inc., Chicago, IL). The level of statistical significance was set at *P* < 0.05. In Experiment 1, only seven pairs of animals exposed to 0.1 mg/kg CZP dose were included in the analysis, as one animal died during the CZP exposure. Due to technical problem during the collection data in Experiment 3, only nine pairs of rats per group were included in the analysis. ANOVA analysis yielded highly similar results for both frequency and duration of behavioral patterns in both Experiment 2 (*social behavior during development*) and 3 (*social behavior in a resident-intruder paradigm*), therefore only data on duration were reported. In addition, we calculated the time in percentage of the occurrence of individual patterns relative to total time spent in social interaction; the values of the most expressed social patterns are given in the results section.

## Results

### Body weight

**Figure 3:**
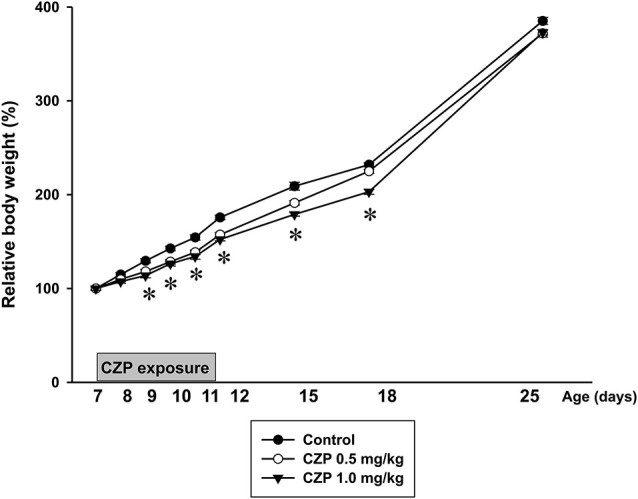
**Body weight (mean +SEM) of rats during CZP exposure and at P12, P15, P18 and P25**. Abscissa: age in days, ordinate: relative weight (body weight on the first day of the drug administration was taken as 100%). * Significant difference compared to appropriate control groups.

There was no difference in average body weight (BW) between litters or between animals selected for individual treatments. BW of P7 animals was 17.8 ± 0.3 g in controls vs. 18.2 ± 0.2 g in CZP 0.5 mg/kg and 18.0 ± 0.3 g in CZP 1.0 mg/kg. CZP exposure had only limited effects on body growth. Controls gained more weight than the CZP exposed rats. Further analysis revealed that from P9 to P12 relative body weight was lower in animals receiving CZP with both doses (0.5 and 1.0 mg/kg) than in controls. In animals exposed to higher dose of CZP (1.0 mg/kg/day) relative body weights were still lower at P15 and P18. No significant differences were found in subsequent days of behavioral testing (i.e., at P25, P32 and P42) [drug effect: *F*_(2,570)_ = 13.34, *p* < 0.001; age *F*_(10,570)_ = 6057.9, *p* < 0.001; drug × age interaction *F*_(20,570)_ = 0.64, *p* = 0.88] (Figure 3).

#### Experiment 1: social behavior during adolescence

*Behavior related to play*. The analysis revealed significant effect of CZP exposure in both the duration *F*_(3,30)_ = 6.50, *P* = 0.002 and the number *F*_(3,30)_ = 7.89, *P* < 0.001 of behavior related to play. At doses of 0.5 and 1.0 mg/kg CZP, *post hoc* comparison showed a significant decrease in the duration and the number of behavior related to play.

**Figure 4:**
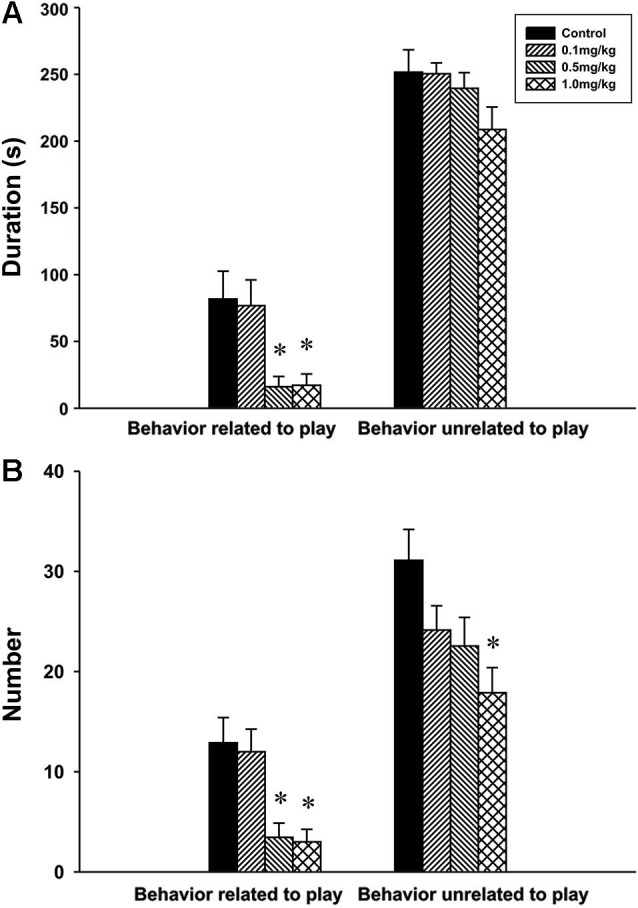
**Duration (A) and number (B) of social behavior related and unrelated to play in P32 animals exposed to CZP from P7 to P11 postnatal day**. * Significant difference compared to control group.

*Behavior unrelated to play*. There was no significant main effect of CZP exposure in the duration of behavior unrelated to play *F*_(3,30)_ = 1.75, *P* = 0.17 but a significant effect was found in the number of behavior unrelated to play *F*_(3,30)_ = 4.11, *P* = 0.01. *Post hoc* test showed that CZP exposure at the dose of 1.0 mg/kg decreased the number of behavior unrelated to play (Figures 4A, B).

#### Experiment 2: social behavior during development

**Figure 5:**
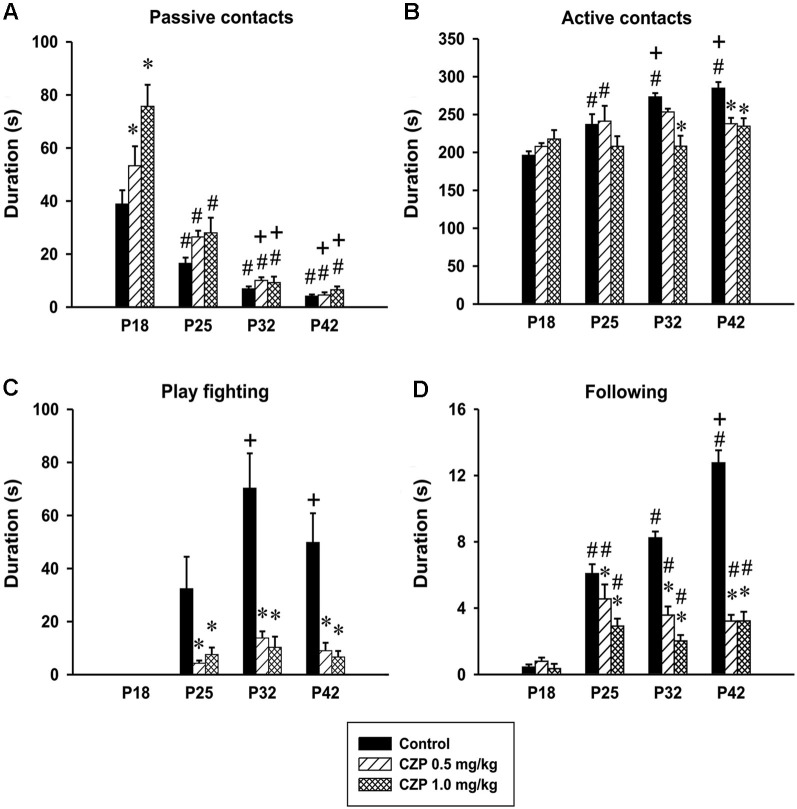
**Effects of CZP exposure (P7–P11) on *social behavior during development***. Illustrated here are the means + SEM for the total duration of passive contacts **(A)**, total duration of active contacts **(B)**, total duration of play fighting **(C)**, and total duration of following **(D)**. * Significant difference compared to appropriate control groups. # Significant difference compared to P18, + Significant difference compared to P25.

*Passive contacts*. There was a significant effect of age in the duration of passive contacts [*F*_(3,81)_ = 99.04, *p* < 0.001]. Both controls and CZP exposed animals spent less time in passive contact at P25, P32 and P42 compared to those at P18. The overall analysis revealed significant effect of CZP exposure [*F*_(2,27)_= 9.60, *p* < 0.001], and drug × age interaction [*F*_(6,81)_ = 4.33, *p* < 0.001]. *Post hoc* comparison showed that both doses of CZP increased passive contact in animals at P18 compared to controls. Further, the animals exposed to CZP at P32 and P42 spent a shorter time in passive contact compared to animals at P25 (Figure 5A).

*Active contacts*. The analysis revealed significant effects of age [*F*_(3,81)_ = 10.78, *p* < 0.001], CZP exposure [*F*_(2,27)_ = 6.79, *P* = 0.004], and drug × age interaction [*F*_(6,81)_ = 4.13, *p* = 0.001]. Control animals at P18 spent significantly less time in active contacts than controls at all subsequent developmental stages. In addition, both controls at P32 and P42 spent more time in active contact compared to those at P25. Furthermore, subsequent analysis showed that P32 animals at the dose of 1.0 mg/kg CZP decreased the time spent in active contact compared to controls. At P42, both doses of CZP decreased the time spent in active contact (Figure 5B).

*Play fighting*. The animals at P18 did not display fighting. Repeated measures of ANOVA revealed significant effects of age [*F*_(3,81)_ = 15.26, *p* < 0.001], drug exposure [*F*_(2,27)_ = 23.31, *p* < 0.001], and drug × age interaction [*F*_(6,81)_ = 6.00, *p* < 0.001]. Control rats at P32 and P42 spent more time fighting compared to those at P25. Both doses of CZP significantly decreased fighting in all age groups (Figure 5C). In addition, the percentage evaluation of the time spent in individual behavioral patterns revealed that CZP exposure irrespective of the dose (0.5 and 1.0 mg) markedly suppressed play fighting at P25 (0.65% and 3.07%), P32 (1.24% and 4.48%) and at P42 (1.23% and 2.65%). The percentage values in the control group are as follows: P25 = 11.09%, P32 = 19.59% and P42 = 14.18%.

*Following*. Finally, the analysis revealed significant effects of age [*F*_(3,81)_=67.99, *p* < 0.001], CZP exposure [*F*_(2,27)_ = 133.62, *p* < 0.001], and drug × age interaction [*F*_(6,81)_ = 22.01, *p* < 0.001] in the time spent following. At P18, a very short duration of following behavior was observed. Subsequent analysis showed that all animals at P25, P32 and P42 irrespective of treatment spent more time following compared to those at P18. In addition, control animals at P42 spent more time following compared to animals at P 25. Moreover, both CZP doses decreased time spent in following behavior at P25, P32 and P42 compared to the appropriate controls (Figure 5D). In addition, both CZP doses suppressed the percentage of time spent in following, above all, at P32 (1.30% and 0.88%) and P42 (1.29% and 1.28%) relative to the control group P32 = 2.30% and P42 = 3.63%.

#### Experiment 3: social behavior of residents in a resident-intruder paradigm

**Figure 6:**
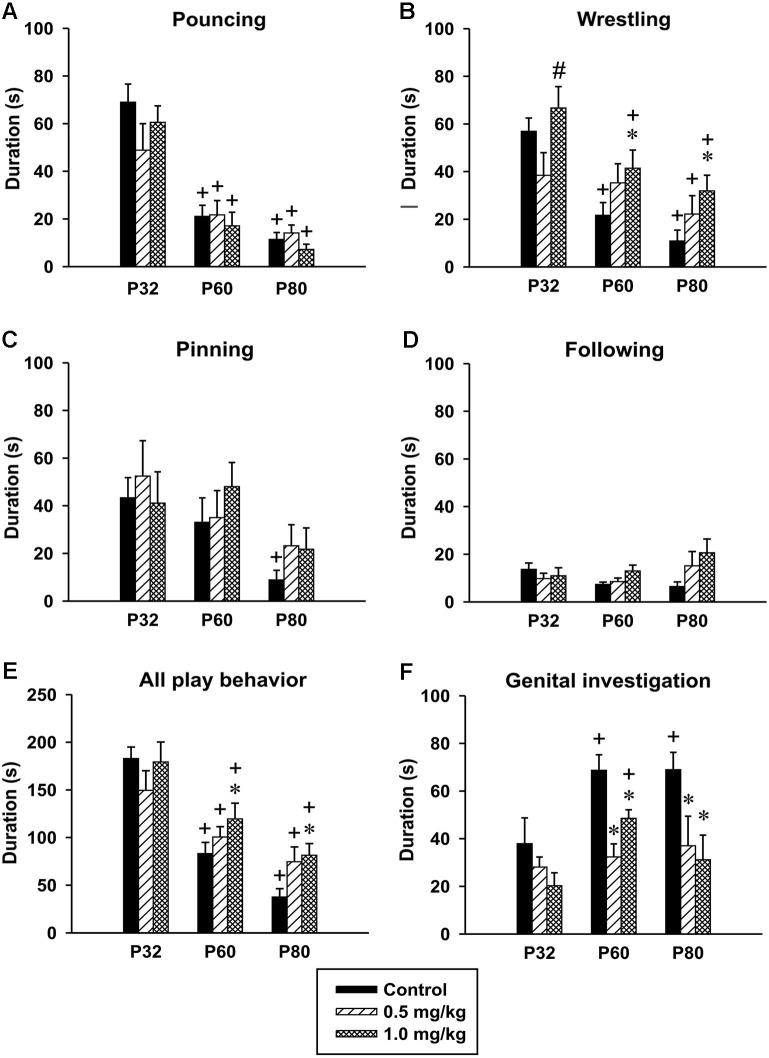
**Effect of CZP exposure (P7–P11) on resident social behavior in a *resident-intruder paradigm***. Illustrated here are the means+SEM for total duration of pouncing **(A)**, total duration of wrestling **(B)**, total duration of pinning **(C)**, total duration of following **(D)**, total duration of (all play behavior) **(E)** and total duration of genital investigation **(F)**. * Significant difference compared to corresponding control groups. + Significant difference compared to P32. # Significant difference compared to 0.5 mg/kg dose of CZP.

*Pouncing*. There was significant effect of age in the time spent pouncing [*F*_(2,48)_ = 55.40, *p* < 0.001]. The animals at both P60 and P80 spent less time pouncing compared to animals at P32. No significant effect of CZP exposure [*F*_(2,24)_ = 0.73, *p* = 0.48] or drug × age interaction was found [*F*_(4,48)_ = 1.24, *p* < 0.30] (Figure 6A).

*Wrestling*. The analysis revealed significant effects of age [*F*_(2,48)_ = 24.93, *p* < 0.001], CZP exposure [*F*_(2,24)_ = 4.97, *p* = 0.01], but not of drug × age interaction [*F*_(4,48)_ = 1.78, *p* = 0.14]. Controls as well as CZP exposed animals spent less time wrestling at P60 and P80 compared to animals at P32. Further, *post hoc* comparison showed that at a dose of 1.0 mg/kg, CZP increased wrestling time in P60 and P80 animals (Figure 6B). The higher dose of CZP increased the percentage of time spent wrestling at P60 = 24.6% and P80 = 28.3% relative to their control groups P60 = 14.3% and P80 = 10.2%.

*Pinning*. There was a significant effect of age [*F*_(2,48)_ = 6.62, *p* < 0.003], but no significant effect of CZP exposure [*F*_(2,24)_ = 0.54, *p* = 0.58], or drug × age interaction *F*_(4,48)_ = 0.49, *p* = 0.73. The *post hoc* analysis showed that only control animals at P80 spent less time pinning compared to those at P32 (Figure 6C).

*Following*. ANOVA failed to identify any significant effect of age [*F*_(2,48)_ = 1.55, *p* = 0.22], CZP exposure [*F*_(2,24)_ = 1.85, *p* = 0.18], and drug × age interaction [*F*_(4,48)_ = 1.98, *p* = 0.1](Figure 6D).

*All play behavior*. Analysis of behavior related to play (pouncing, wrestling, pinning and following) revealed significant age effect [*F*_(2,48)_ = 39.40, *p* < 0.001]. Both controls and CZP exposed animals at P60 and P80 spent less time playing compared to those at P32. ANOVA did not revealed any significant effects of CZP exposure [*F*_(2,24)_ = 2.31, *p* = 0.12], as well as drug × age interaction [*F*_(4,48)_ = 1.58, *p* = 0.19]. Nevertheless, *post hoc* comparison showed that animals exposed to CZP at a dose of 1.0 mg/kg spent more time playing compared to their respective controls (Figure 6E).

*Genital investigation*. There was significant effect of age in the time spent investigating genitals [*F*_(2,48)_ = 6.13, *p* = 0.004]. Control animals at P60 and P80 spent more time investigating genitals compared to those at P32. Both doses of CZP affected genital investigation [*F*_(2,24)_ = 11.41, *p* < 0.001]. *Post hoc* test revealed that at both P60 and P80 the animals spent less time investigating genitals than the corresponding controls. Animals exposed to 1.0 mg/kg CZP at P60 spent more time investigating, compared to those at P32. No drug × age interaction effect was found [*F*_(2,48)_ = 1.37, *p* = 0.26] (Figure 6F). As for the percentage of time spent in genital investigation, both CZP doses markedly suppressed this behavior at P60 (24.3% and 24.3%) and P80 (33.2% and 27.7%) relative to control groups (P60 = 45.2% and P80 = 64.7%).

### Locomotion

**Figure 7:**
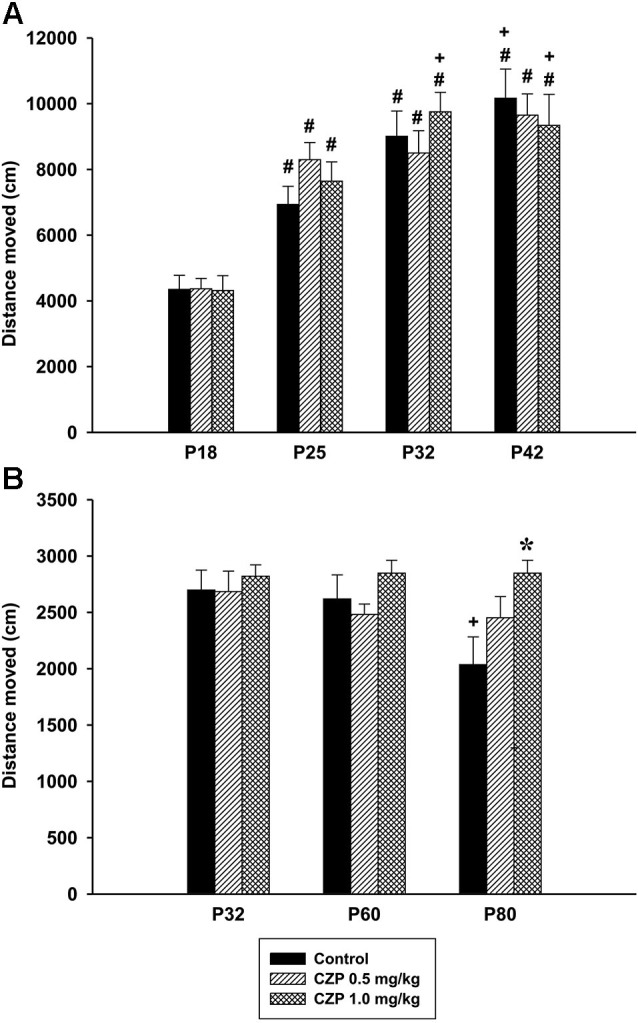
**Effect of CZP exposure (P7–P11) on locomotion**. Illustrated here are the data from Experiment 2 (*social behavior during development, **A***) and Experiment 3 (*social behavior in a resident-intruder paradigm, **B***). # Significant difference compared to P18. + Significant difference compared to P25. * Significant difference compared to control group.

In Experiment 1 (*social behavior during adolescence*) ANOVA did not reveal significant difference in the distance moved between the control and CZP animals, aged 32 days (control: mean = 8215.56, SEM = 445.50, CZP 0.1 mg/kg: mean = 8128.01, SEM = 787.61, CZP 0.5 mg/kg: mean = 7560.67, SEM = 737.79, CZP 1.0 mg/kg: mean = 8123.96, SEM = 445.557). During development (Experiment 2), there was a significant effect of age [*F*_(3,81)_ = 71.71, *p* < 0.001], but not of CZP exposure [*F*_(2,27)_ = 0.24, *p* = 0.97], nor of drug × age interaction [*F*_(6,81)_ = 1.40, *p* = 0.22]. *Post hoc* test revealed that animals in all age groups walked a longer distance than those at P18. The dose of 1.0 mg/kg CZP significantly prolonged the distance moved at P32 and P42 compared to those of animals at P25 (Figure 7A). As for locomotion of resident rats, ANOVA revealed a significant effect of age [*F*_(2,48)_ = 4.20, *p* = 0.021] and drug × age interaction [*F*_(4,48)_ = 2.57 *p* = 0.04]. No significant effect of CZP exposure [*F*_(2,24)_ = 2.38, *p* = 0.11] was observed in the distance moved. *Post hoc* test showed a significant decline in locomotion in control animals at P80 compared to those at P32 and P60. Moreover, the animals at P80 exposed to a higher CZP dose, walked for a longer distance compared to respective controls (Figure 7B).

## Discussion

In the present study, we evaluated the effects of early life exposure to clonazepam on the social behavior of rats in respect to their familiarity with test conditions. The results of this study have shown that short-lasting exposure to CZP during early postnatal period affects social play and social interaction differentially, depending on age and environment.

Social behavior represents a relevant category of behavior essential for the acquisition of motor, social, sexual and cognitive skills (Vanderschuren et al., 1997; Pellis and Pellis, 1998; Auger and Olesen, 2009; Trezza et al., 2010). In rats, social behavior is a complex dynamic interaction that begins with social play behavior at about P18, peaks between P32 and P40 and gradually declines thereafter (Panksepp, 1981; Pellis and Pellis, 1997; Terranova et al., 1999) but never disappears completely (Vanderschuren et al., 1995a; Pellis and Pellis, 1997). Social interactions and play behavior especially are highly dependent not only on the age of rats, but also on test circumstances, primarily on the familiarity or unfamiliarity of the environment (Vanderschuren et al., 1995b; Varlinskaya and Spear, 2008). Novel environment represents a highly anxiogenic condition and novelty was found to suppress social interaction in pubescent and young adult animals (P35 and P60) but not in younger animals (P28) (Primus and Kellogg, 1989, 1990).

Many experimental studies already documented that social behavior is particularly sensitive to pharmacological influences (Varlinskaya and Spear, 2002; File and Seth, 2003; Schneider and Koch, 2005; Homberg et al., 2007; Koros et al., 2007; Trezza et al., 2009; Mooney and Varlinskaya, 2011). Also early pharmacological intervention was found to modify social behavior long time after treatment cessation. File (1986b) reported minor increase of social interactions between control adolescent rats and rats treated with diazepam or lorazepam throughout preweaning period, but no difference in animals treated with CZP according to the same treatment protocol (File, 1986c). In both studies, animals spent more time in active social interaction when being in familiar than in unfamiliar environment. On the contrary, in our study CZP exposure suppressed play behavior in adolescence, but not other forms of social interaction irrespective of environmental context. Discrepancy between these studies probably relates to differences in experimental protocol and way of evaluation. In our study, we differentiated between “*social behavior related to play”* and “*social behavior unrelated to play”* and these two categories were evaluated separately, whereas File evaluated all social contacts in one category. Thus we hypothesize that early CZP exposure leads to increased social anxiety and/or decreased motivation for play behavior and that the lack of familiarity effects is due to impaired adaptation to novelty. The adolescent animals were familiarized to OF before social test. In contrast, in developmental study animals were not habituated to environment. Pre-weaning rats do not display intra-session habituation and even older rats are not able to remember the environment if exposure is repeated after seven days from the first exposure (Leussis and Bolivar, 2006).

In present study, adolescent animals were isolated for three hours before the social interaction took place in order to promote social behavior (Vanderschuren et al., 1995b). It has been shown that social deprivation, especially during the acquisition period of social play, leads to social disturbances independent of subsequent social stimulation (Hol et al., 1999) and may induce a predisposition to either anxiety or depressive-like behavior (Tsoory et al., 2007). Developing animals were repeatedly tested thus the time period of isolation was cut to 30 min. Therefore we assume that the inhibition of social play in CZP-exposed animals cannot be attributed to a long-term or repeated isolation.

When allowed to establish a territory, a male rat defends his territory if an unfamiliar conspecific intruder is introduced. Such behavior is indicative of the territorial advantage of resident animal, and it is apparent after the resident has been singly housed for only a few days (for rev. Mitchell and Redfern, 2005). In concordance with previous studies on the role of territoriality on social behavior in a resident-intruder paradigm, our data show that play behavior gradually decreases with age but never disappear completely and even adult rats continue to play (Pellis and Pellis, 1987, 1990). Early exposure to CZP modified social interaction in resident-intruder test. Exposure to CZP in a dose of 1.0 mg/kg increased the duration of wrestling which was always initiated by the resident rat reflecting a high degree of dominance for his territory. This social activity never changed to an aggressive form of fighting characterized by a threat posture, serious attacks to partner’s rump, and biting (Pellis and Pellis, 1987; Blanchard et al., 2001). This finding is in agreement with results of previous study showing that if a male intruder is weight-matched to resident, the aggressive form of interaction does not emerge (Robinson et al., 2011). In earlier studies, File reported that the administration of CZP from birth until weaning enhances offensive behavior when adolescent rats were resident in their home cage (File, 1986c). Similarly, the same author showed that neonatal treatment (P1–P7) with a high dose of diazepam (10 mg/kg) increases aggression in adolescent resident rats (File, 1987). Taken together, these findings indicate that BZDs exposure during postnatal period affects the specific forms of social behavior that reflect dominance for an established territory. Our data in addition revealed a remarkable inhibition of genital investigation in the adults exposed to CZP early in life. This component of social behavior represents a natural propensity of laboratory rats to investigate and olfactory discriminate conspecifics (Engelmann et al., 1995, 2011). Thus present findings suggest that postnatal CZP exposure shifts the behavior from social investigation to a higher motivation pattern, such as play wrestling. Opposite effects of early CZP exposure on social play and other forms of social behavior are consistent with recent studies demonstrating that social play and social behaviors represent separate behavioral categories with different pharmacological sensitivity (Varlinskaya et al., 1999; Varlinskaya and Spear, 2008; Trezza et al., 2009).

Early CZP exposure did not result in substantial changes of locomotion in any social test. Therefore we consider it unlikely that the differences in social behavior are related to locomotor alteration. Exposure to the high dose of CZP however resulted in decrease of capability to adapt to environmental condition. In contrast to controls, in CZP exposed animals distance moved did not decrease with repeated exposure. As previously reported animals exposed to CZP exhibited higher locomotion, spent more time in the central part of the OF, suggesting decresed anxiety, and had impaired between-session habituation in (Mikulecká et al., 2014).

The expression of social behavior and specifically play behavior involves a wide variety of neuronal systems. The various aspects of social play are evidently regulated and/or modulated by different neurotransmitters and can be influenced differently by environmental and social factors (Varlinskaya et al., 1999; Trezza et al., 2009). The recent progress in the neuropharmacology of play behavior have pointed to neurotransmitter systems, such as opioids, cannabinoids, cholinergic and dopaminergic that modulate the rewarding, motivational and cognitive aspects of play behavior, and to the nonspecific effects of serotonin and norepinephrine (Vanderschuren et al., 1997; Homberg et al., 2007; Trezza et al., 2010; Siviy and Panksepp, 2011; Siviy et al., 2011). In addition, GABAergic and dopaminergic transmission play a major role in the pharmacology, neurochemistry and physiopathology of the emotional states. For example, GABA_A_ receptor substrate which controls bidirectional reward signaling between dopaminergic and non-dopaminergic reward systems, play a role in positive emotional processes such as social play (Laviolette and van der Kooy, 2001; Burgdorf and Panksepp, 2006).

Interactive social play represents a separate and characteristic form of social behavior expressed predominantly between weaning and adolescence. This form of social behavior is highly rewarding and indispensable for the development of social competence and cognitive skills (Panksepp, 1981; Pellis and Mckenna, 1995). Social play during a juvenile period prepares the motor system of animals for engagement in adult behavior. In addition, the experience of positive social interaction during key developmental ages has profound and long-lasting effects on brain function and behavior in emotional, motivational and cognitive domain (Trezza et al., 2011). When available, play can also facilitate and refine brain areas that are involved in the very social skills, and serves for training animals to cope with unpredictable events. Play deprivation during juvenile and adolescence can lead to incompetence in sexual performance in adulthood and produce long-term cognitive, behavioral and emotional deficits (for rev. Pellis and Pellis, 2006; Trezza et al., 2011). Short-term CZP exposure in the critical period of neurobehavioral development may interfere with the formation of social motivation or positive emotional processes, thereby altering the emotional reactivity and expression of social behavior. Inadequate social behavior is the main hallmark of psychiatric disorders (DMS IV, American Psychiatric Association, 1994) and to some extent, the disturbances shown in animal studies are comparable to symptomatological as well as etiological aspects of neurodevelopmental disorders (Schneider and Koch, 2005). The data from animal experiments have to be translated to human situation with extreme caution. In concordance with previous studies, our results however indicate that the exposure to benzodiazepines during the critical developmental period may alter the responsiveness in social relationship.

## Conflict of interest statement

The authors declare that the research was conducted in the absence of any commercial or financial relationships that could be construed as a potential conflict of interest.

## References

[B1] American Psychiatric Association. (1994). Diagnostic and Statistical Manual of Mental Disorders. Washington, DC: American Psychiatric Press

[B2] André M. Boutroy M. J. Dubruc C. Thenot J. P. Bianchetti G. Sola L. (1986). Clonazepam pharmacokinetics and therapeutic efficacy in neonatal seizures. Eur. J. Clin. Pharmacol. 30, 585–589 10.1007/bf00542419 3758147

[B3] Auger A. P. Olesen K. M. (2009). Brain sex differences and the organisation of juvenile social play behaviour. J. Neuroendocrinol. 21, 519–525 10.1111/j.1365-2826.2009.01871.x 19500222 PMC2739448

[B4] Avishai-Eliner S. Brunson K. L. Sandman C. A. Baram T. Z. (2002). Stressed-out, or in (utero)? Trends Neurosci. 25, 518–524 10.1016/s0166-2236(02)02241-5 12220880 PMC2930786

[B5] Bittigau P. Sifringer M. Genz K. Reith E. Pospischil D. Govindarajalu S. (2002). Antiepileptic drugs and apoptotic neurodegeneration in the developing brain. Proc. Natl. Acad. Sci. U S A 99, 15089–15094 10.1073/pnas.222550499 12417760 PMC137548

[B6] Blanchard R. J. Yudko E. Dulloog L. Blanchard D. C. (2001). Defense changes in stress nonresponsive subordinate males in a visible burrow system. Physiol. Behav. 72, 635–642 10.1016/s0031-9384(00)00449-2 11336994

[B7] Burgdorf J. Panksepp J. (2006). The neurobiology of positive emotions. Neurosci. Biobehav. Rev. 30, 173–187 10.1016/j.neubiorev.2005.06.001 16099508

[B8] Clancy B. Finlay B. L. Darlington R. B. Anand K. J. (2007). Extrapolating brain development from experimental species to humans. Neurotoxicology 28, 931–937 10.1016/j.neuro.2007.01.014 17368774 PMC2077812

[B9] Darbra S. Pallarès M. (2009). Neonatal allopregnanolone increases novelty-directed locomotion and disrupts behavioural responses to GABA(A) receptor modulators in adulthood. Int. J. Dev. Neurosci. 27, 617–625 10.1016/j.ijdevneu.2009.05.008 19481145

[B10] Dobbing J. Sands J. (1979). Comparative aspects of the brain growth spurt. Early. Hum. Dev. 3, 79–83 10.1016/0378-3782(79)90022-7 118862

[B11] Engelmann M. Hadicke J. Noack J. (2011). Testing declarative memory in laboratory rats and mice using the nonconditioned social discrimination procedure. Nat. Protoc. 6, 1152–1162 10.1038/nprot.2011.353 21799485

[B12] Engelmann M. Wotjak C. T. Landgraf R. (1995). Social discrimination procedure: an alternative method to investigate juvenile recognition abilities in rats. Physiol. Behav. 58, 315–321 10.1016/0031-9384(95)00053-l 7568435

[B13] File S. E. (1986a). Behavioral changes persisting into adulthood after neonatal benzodiazepine administration in the rat. Neurobehav. Toxicol. Teratol. 8, 453–461 2878379

[B14] File S. E. (1986b). Effects of neonatal administration of diazepam and lorazepam on performance of adolescent rats in tests of anxiety, aggression, learning and convulsions. Neurobehav. Toxicol. Teratol. 8, 301–306 2874510

[B15] File S. E. (1986c). The effects of neonatal administration of clonazepam on passive avoidance and on social, aggressive and exploratory behavior of adolescent male rats. Neurobehav. Toxicol. Teratol. 8, 447–452 3785507

[B16] File S. E. (1987). Diazepam and caffeine administration during the first week of life: changes in neonatal and adolescent behavior. Neurotoxicol. Teratol. 9, 9–16 10.1016/0892-0362(87)90063-8 3627081

[B17] File S. E. Seth P. (2003). A review of 25 years of the social interaction test. Eur. J. Pharmacol. 463, 35–53 10.1016/s0014-2999(03)01273-1 12600701

[B18] Forcelli P. A. Kozlowski R. Snyder C. Kondratyev A. Gale K. (2012). Effects of neonatal antiepileptic drug exposure on cognitive, emotional and motor function in adult rats. J. Pharmacol. Exp. Ther. 340, 558–566 10.1124/jpet.111.188862 22129597 PMC3286323

[B19] Fraňková S. Mikulecká A. (1990). Ontogeny of social behavior of pups of laboratory rats genetically selected for activity level. Act. Nerv. Super. (Praha) 32, 167–173 2260416

[B20] Gai N. Grimm V. E. (1982). The effect of prenatal exposure to diazepam on aspects of postnatal development and behavior in rats. Psychopharmacology (Berl) 78, 225–229 10.1007/bf00428155 6818579

[B21] Garrett K. M. Saito N. Duman R. S. Abel M. S. Ashton R. A. Fujimori S. (1990). Differential expression of gamma-aminobutyric acidA receptor subunits. Mol. Pharmacol. 37, 652–657 2160058

[B22] Hol T. Van den Berg C. L. Van Ree J. M. Spruijt B. M. (1999). Isolation during the play period in infancy decreases adult social interactions in rats. Behav. Brain Res. 100, 91–97 10.1016/s0166-4328(98)00116-8 10212056

[B23] Homberg J. R. Schiepers O. J. Schoffelmeer A. N. Cuppen E. Vanderschuren L. J. (2007). Acute and constitutive increases in central serotonin levels reduce social play behaviour in peri-adolescent rats. Psychopharmacology (Berl) 195, 175–182 10.1007/s00213-007-0895-8 17661017 PMC2048539

[B24] Hoogerkamp A. Arends R. H. Bomers A. M. Mandema J. W. Voskuyl R. A. Danhof M. (1996). Pharmacokinetic/pharmacodynamic relationship of benzodiazepines in the direct cortical stimulation model of anticonvulsant effect. J. Pharmacol. Exp. Ther. 279, 803–812 8930187

[B25] Hulin M. W. Amato R. J. Winsauer P. J. (2012). GABA(A) receptor modulation during adolescence alters adult ethanol intake and preference in rats. Alcohol. Clin. Exp. Res. 36, 223–233 10.1111/j.1530-0277.2011.01622.x 21895721 PMC3234308

[B26] Kellogg C. K. (1988). Benzodiazepines: influence on the developing brain. Prog. Brain Res. 73, 207–228 10.1016/s0079-6123(08)60506-3 2843947

[B27] Koros E. Rosenbrock H. Birk G. Weiss C. Sams-Dodd F. (2007). The selective mGlu5 receptor antagonist MTEP, similar to NMDA receptor antagonists, induces social isolation in rats. Neuropsychopharmacology 32, 562–576 10.1038/sj.npp.1301133 16794564

[B28] Kubová H. Mareš P. (1989). Time course of the anticonvulsant action of clonazepam during ontogenesis in the rat. Arch. Int. Pharmacodyn. Ther. 298, 15–24 2757463

[B29] Lader M. (1994). Biological processes in benzodiazepine dependence. Addiction 89, 1413–1418 10.1111/j.1360-0443.1994.tb03737.x 7841850

[B30] Lalive A. L. Rudolph U. Luscher C. Tan K. R. (2011). Is there a way to curb benzodiazepine addiction? Swiss Med. Wkly. 141:w13277 10.4414/smw.2011.13277 22012428

[B31] Laviolette S. R. van der Kooy D. (2001). GABA(A) receptors in the ventral tegmental area control bidirectional reward signalling between dopaminergic and non-dopaminergic neural motivational systems. Eur. J. Neurosci. 13, 1009–1015 10.1046/j.1460-9568.2001.01458.x 11264674

[B32] Leussis M. P. Bolivar V. J. (2006). Habituation in rodents: a review of behavior, neurobiology, and genetics. Neurosci. Biobehav. Rev. 30, 1045–1064 10.1016/j.neubiorev.2006.03.006 16774787

[B33] Lippa A. S. Beer B. Sano M. C. Vogel R. A. Meyerson L. R. (1981). Differential ontogeny of type 1 and type 2 benzodiazepine receptors. Life Sci. 28, 2343–2347 10.1016/0024-3205(81)90498-7 6265728

[B34] Meaney M. Stewart J. (1981). A descriptive study of social development in the rat (*Rattus norvegicus*). Anim. Behav. 29, 34–45 10.1016/s0003-3472(81)80149-2

[B35] Mikulecká A. Mareš P. Kubová H. (2011). Rebound increase in seizure susceptibility but not isolation-induced calls after single administration of clonazepam and Ro 19–8022 in infant rats. Epilepsy Behav. 20, 12–19 10.1016/j.yebeh.2010.10.021 21130691

[B36] Mikulecká A. Šubrt M. Stuchlík A. Kubová H. (2014). Consequences of early postnatal benzodiazepines exposure in rats. I. Cognitive-like behavior. Front. Behav. Neurosci. 8:101 10.3389/fnbeh.2014.00101 24734010 PMC3975106

[B37] Mitchell P. J. Redfern P. H. (2005). Animal models of depressive illness: the importance of chronic drug treatment. Curr. Pharm. Des. 11, 171–203 10.2174/1381612053382250 15638757

[B68] Mòdol L. Darbra S. Vallèe M. Pallarès M. (2013). Alteration of neonatal Allopregnanolone levels affects exploration, anxiety, aversive learning and adult behavioural response to intrahippocampal neurosteroids. Behav. Brain Res. 241, 96–104 10.1016/j.bbr.2012.11.043 23228522

[B38] Mooney S. M. Varlinskaya E. I. (2011). Acute prenatal exposure to ethanol and social behavior: effects of age, sex and timing of exposure. Behav. Brain Res. 216, 358–364 10.1016/j.bbr.2010.08.014 20728475 PMC2975787

[B39] Morishita S. (2009). Clonazepam as a therapeutic adjunct to improve the management of depression: a brief review. Hum. Psychopharmacol. 24, 191–198 10.1002/hup.1015 19330803

[B40] Panksepp J. (1981). The ontogeny of play in rats. Dev. Psychobiol. 14, 327–332 10.1002/dev.420140405 7250521

[B41] Pellis S. M. Mckenna M. (1995). What do rats find rewarding in play fighting—an analysis using drug-induced non-playful partners. Behav. Brain Res. 68, 65–73 10.1016/0166-4328(94)00161-8 7619307

[B42] Pellis S. M. Pellis V. C. (1987). Play fighting differs from serious fighting in both target of attack and tactics of fighting in the laboratory rat (*Rattus norvegicus*). Aggress. Behav. 13, 227–242 10.1002/1098-2337(1987)13:4<227::aid-ab2480130406>3.0.co;2-c

[B46] Pellis S. M. Pellis V. C. (1990). Differential rates of attack, defense and counterattack during the developmental decrease in play fighting by male and female rats. Dev. Psychobiol. 23, 215–231 10.1002/dev.420230303 2379760

[B44] Pellis S. M. Pellis V. C. (1997). The prejuvenile onset of play fighting in laboratory rats (*Rattus norvegicus*). Dev. Psychobiol. 31, 193–205 10.1002/(sici)1098-2302(199711)31:3<193::aid-dev4>3.3.co;2-# 9386921

[B45] Pellis S. M. Pellis V. C. (1998). Play fighting of rats in comparative perspective: a schema for neurobehavioral analyses. Neurosci. Biobehav. Rev. 23, 87–101 10.1016/s0149-7634(97)00071-7 9861614

[B43] Pellis S. M. Pellis V. C. (2006). “Play and the development of social engagement: a comparative perspective,” in The Development of Social Engagement: Neurobiological Perspectives, eds Marshall P. J. Fox N. A. (New York: Oxford University Press), 247–274

[B47] Primus R. J. Kellogg C. K. (1989). Pubertal-related changes influence the development of environment-related social interaction in the male rat. Dev. Psychobiol. 22, 633–643 10.1002/dev.420220608 2792573

[B48] Primus R. J. Kellogg C. K. (1990). Developmental influence of gonadal function on the anxiolytic effect of diazepam on environment-related social interaction in the male rat. Behav. Pharmacol. 1, 437–446 10.1097/00008877-199000150-00005 11175428

[B49] Raol Y. H. Zhang G. Budreck E. C. Brooks-Kayal A. R. (2005). Long-term effects of diazepam and phenobarbital treatment during development on GABA receptors, transporters and glutamic acid decarboxylase. Neuroscience 132, 399–407 10.1016/j.neuroscience.2005.01.005 15802192

[B50] Robinson D. L. Zitzman D. L. Smith K. J. Spear L. P. (2011). Fast dopamine release events in the nucleus accumbens of early adolescent rats. Neuroscience 176, 296–307 10.1016/j.neuroscience.2010.12.016 21182904 PMC3061289

[B51] Schneider M. Koch M. (2005). Deficient social and play behavior in juvenile and adult rats after neonatal cortical lesion: effects of chronic pubertal cannabinoid treatment. Neuropsychopharmacology 30, 944–957 10.1038/sj.npp.1300634 15592349

[B52] Schroeder H. Humbert A. C. Desor D. Nehlig A. (1997). Long-term consequences of neonatal exposure to diazepam on cerebral glucose utilization, learning, memory and anxiety. Brain Res. 766, 142–152 10.1016/s0006-8993(97)00538-6 9359597

[B53] Siviy S. M. Crawford C. A. Akopian G. Walsh J. P. (2011). Dysfunctional play and dopamine physiology in the Fischer 344 rat. Behav. Brain Res. 220, 294–304 10.1016/j.bbr.2011.02.009 21335036 PMC3081852

[B54] Siviy S. M. Panksepp J. (2011). In search of the neurobiological substrates for social playfulness in mammalian brains. Neurosci. Biobehav. Rev. 35, 1821–1830 10.1016/j.neubiorev.2011.03.006 21414353

[B55] Smart J. L. Dobbing J. Adlard B. P. Lynch A. Sands J. (1973). Vulnerability of developing brain: relative effects of growth restriction during the fetal and suckling periods on behavior and brain composition of adult rats. J. Nutr. 9, 1327–1338 4725720 10.1093/jn/103.9.1327

[B56] Terranova M. L. Cirulli F. Laviola G. (1999). Behavioral and hormonal effects of partner familiarity in periadolescent rat pairs upon novelty exposure. Psychoneuroendocrinology 24, 639–656 10.1016/s0306-4530(99)00019-0 10399773

[B57] Trezza V. Baarendse P. J. Vanderschuren L. J. (2009). Prosocial effects of nicotine and ethanol in adolescent rats through partially dissociable neurobehavioral mechanisms. Neuropsychopharmacology 34, 2560–2573 10.1038/npp.2009.85 19657330 PMC2774531

[B58] Trezza V. Baarendse P. J. Vanderschuren L. J. (2010). The pleasures of play: pharmacological insights into social reward mechanisms. Trends Pharmacol. Sci. 31, 463–469 10.1016/j.tips.2010.06.008 20684996 PMC2946511

[B59] Trezza V. Campolongo P. Vanderschuren L. J. (2011). Evaluating the rewarding nature of social interactions in laboratory animals. Dev. Cogn. Neurosci. 1, 444–458 10.1016/j.dcn.2011.05.007 22436566 PMC6987553

[B60] Tsoory M. Cohen H. Richter-Levin G. (2007). Juvenile stress induces a predisposition to either anxiety or depressive-like symptoms following stress in adulthood. Eur. Neuropsychopharmacol. 17, 245–256 10.1016/j.euroneuro.2006.06.007 16889944

[B61] Tucker J. C. (1985). Benzodiazepines and the developing rat: a critical review. Neurosci. Biobehav. Rev. 9, 101–111 10.1016/0149-7634(85)90036-3 2858075

[B62] Vanderschuren L. J. Niesink R. J. Spruijt B. M. van Ree J. M. (1995a). Effects of morphine on different aspects of social play in juvenile rats. Psychopharmacology (Berl) 117, 225–231 10.1007/bf02245191 7753971

[B63] Vanderschuren L. J. Niesink R. J. Spruijt B. M. van Ree J. M. (1995b). Influence of environmental factors on social play behavior of juvenile rats. Physiol. Behav. 58, 119–123 10.1016/0031-9384(94)00385-i 7667408

[B64] Vanderschuren L. J. Niesink R. J. van Ree J. M. (1997). The neurobiology of social play behavior in rats. Neurosci. Biobehav. Rev. 21, 309–326 10.1016/s0149-7634(96)00020-6 9168267

[B65] Varlinskaya E. I. Spear L. P. (2002). Acute effects of ethanol on social behavior of adolescent and adult rats: role of familiarity of the test situation. Alcohol. Clin. Exp. Res. 26, 1502–1511 10.1111/j.1530-0277.2002.tb02449.x 12394283

[B66] Varlinskaya E. I. Spear L. P. (2008). Social interactions in adolescent and adult Sprague-Dawley rats: impact of social deprivation and test context familiarity. Behav. Brain Res. 188, 398–405 10.1016/j.bbr.2007.11.024 18242726 PMC3057041

[B67] Varlinskaya E. I. Spear L. P. Spear N. E. (1999). Social behaviour and social motivation in adolescent rats: role of housing conditions and partner’s activity. Physiol. Behav. 67, 475–482 10.1016/s0031-9384(98)00285-6 10549884

